# M2 receptor activation inhibits cell cycle progression and survival in human glioblastoma cells

**DOI:** 10.1111/jcmm.12038

**Published:** 2013-03-14

**Authors:** Michela Ferretti, Cinzia Fabbiano, Maria Di Bari, Claudia Conte, Emilia Castigli, Miriam Sciaccaluga, Donatella Ponti, Paola Ruggieri, Antonino Raco, Ruggero Ricordy, Antonella Calogero, Ada Maria Tata

**Affiliations:** a Department of Biology and Biotechnologies Charles Darwin, Research Centre of Neurobiology Daniel Bovet, La Sapienza, University of RomeP.le Aldo Moro, Roma, Italy; b Institute of Molecular Biology and Pathology, CNRRome, Italy; c Department of Medico-Surgical Sciences and Biotechnologies, La Sapienza, University of RomeLatina, Italy; d Department of Cellular and Environmental Biology, University of PerugiaPerugia, Italy; e UOC Neurosurgery, S. Andrea Hospital, University of RomeSapienza, Italy

**Keywords:** apoptosis, arecaidine, cell cycle, glioblastoma, M2 muscarinic receptors, proliferation

## Abstract

Muscarinic receptors, expressed in several primary and metastatic tumours, appear to be implicated in their growth and propagation. In this work we have demonstrated that M2 muscarinic receptors are expressed in glioblastoma human specimens and in glioblastoma cell lines. Moreover, we have characterized the effects of the M2 agonist arecaidine on cell growth and survival both in two different glioblastoma cell lines (U251MG and U87MG) and in primary cultures obtained from different human biopsies. Cell growth analysis has demonstrated that the M2 agonist arecaidine strongly decreased cell proliferation in both glioma cell lines and primary cultures. This effect was dose and time dependent. FACS analysis has confirmed cell cycle arrest at G1/S and at G2/M phase in U87 cells and U251 respectively. Cell viability analysis has also shown that arecaidine induced severe apoptosis, especially in U251 cells. Chemosensitivity assays have, moreover, shown arecaidine and temozolomide similar effects on glioma cell lines, although IC50 value for arecaidine was significantly lower than temozolomide. In conclusion, we report for the first time that M2 receptor activation has a relevant role in the inhibition of glioma cell growth and survival, suggesting that M2 may be a new interesting therapeutic target to investigate for glioblastoma therapy.

## Introduction

Acetylcholine (ACh) is a well-known signalling molecule widely distributed in several organisms and tissues 1. In addition to its function as a neurotransmitter, ACh modulates cell survival, proliferation and differentiation in neuronal and non-neuronal cells 2–4. In fact it has been demonstrated that muscarinic and nicotinic acetylcholine receptors are expressed in oligodendrocytes and their precursors 2, 3, 5, 6, in Schwann cells 7, and in neural progenitors 8, 9.

Furthermore, muscarinic receptors are involved in several neurological disorders such as schizophrenia (M5 receptor), Alzheimer's (AD; M1 receptor) and Parkinson's diseases (M4 receptor). New muscarinic agonists (*e.g*. alvameline, xanomeline) have been recently considered for treatment of these neuropathologies; some of these agonists are already in use or under clinical trials 10–12.

Muscarinic acetylcholine receptors (mAChRs) belong to the family of seven helix transmembrane receptors coupled to G-Proteins; five muscarinic subtypes have been cloned in different animal species. They have a high degree of homology, showing significant variability in specific regions, such as the carboxy- and amino- terminals and the third cytoplasmic loop 13. They activate several transduction pathways, some of which are involved in the control of cell proliferation (*e.g*. IP3K, MAPK/ERK kinases) 14.

mAChRs are expressed in several primary and metastatic tumours such as colon, ovary, prostate, lung carcinomas 15–19, breast cancer and melanoma 20–23. In some cases ACh, synthesized by the tumour cells, modulates cell proliferation by an autocrine mechanism that involves cholinergic receptors of nicotinic and muscarinic types 19. Although a direct or indirect involvement of transduction pathways activated by cholinergic receptors has not been demonstrated, either the inhibition of ACh synthesis and release or the use of nicotinic and muscarinic antagonists are able to counteract tumour cell growth and slow the tumour progression in small lung carcinoma 24. In mammary adenocarcinoma and melanoma cell lines, mAChRs can also modulate cell migration and angiogenesis, suggesting the possible involvement also in the metastases formation 20, 21, 23.

Although muscarinic receptors have been identified in normal glial cells 2, 3, 5, 6, 25 and in low-grade astrocytoma 26, the characterization of mAChR effects on more aggressive brain tumours such as grade IV glioblastoma (GBM) has not yet been investigated.

Glioblastomas are the most common brain tumours in humans. Average survival of patients affected by glioblastoma ranges from 6 months to 2 years. Although advances in the chemotherapeutic treatment of glioblastoma (GBM) have been made, almost 80% of the patients die within 2 years after diagnosis, underlining the need of new agents to improve chemotherapy protocols. The cell heterogeneity of glioma complicates the chemotherapeutic response, on the basis of the differential chemosensitivity of tumour cells to conventional pharmacological agents 27. Recently we demonstrated that the selective stimulation of M2 acetylcholine receptors in glioma cell lines caused a decrease in cell proliferation 28.

On the basis of these considerations, we have further assessed the role of M2 muscarinic receptors in the proliferation and survival of glioblastoma cells *in vitro*. Here we report the results of our studies on M2 receptor distribution and the effects of the M2 agonist arecaidine on cell cycle progression and survival in two established glioma cell lines and in tumour biopsy primary cultures. We have also included a comparative evaluation of the cytotoxic effects of arecaidine and temozolomide. Finally, the use of specific muscarinic antagonists and the silencing of the M2 receptor have been performed to directly demonstrate the involvement of M2 receptor in the inhibition of glioma cell proliferation.

## Materials and methods

### Cell cultures

The human glioblastoma cell lines used in this study were U87MG, presenting a deletion of the tumour suppressor gene PTEN, and the U251MG, characterized by mutations of the p53 gene. The cells were grown in DMEM supplemented with 1% non-essential amino acids (Sigma-Aldrich, St. Louis, MO, USA), 50 μg/ml streptomycin, 50 IU/ml penicillin, 2 mM glutamine (Sigma-Aldrich) and 10% or 0.5% foetal calf serum (FCS; Sigma-Aldrich).

The preparation of primary cell cultures from biopsies, their maintenance *in vitro* and pharmacological characterization have been previously described 29.

### Muscarinic agonist and antagonist treatments

Cells were incubated in presence of the M2 selective agonist arecaidine (final concentration 10 and 100 μM) 25 for different times of treatment (24, 48, 72 and 96 hrs). Arecaidine is an alkaloid extracted from areca nut. It displays several different pharmacological effects (*e.g*. digestant and vermifuge) 30. The alkaloids of the areca nut bind cholinergic receptors and in particular arecaidine has been demonstrated to have specific agonist property for M2 muscarinic receptor 31. To confirm the involvement of M2 receptor subtype in our experiments, several muscarinic antagonists were used such as: gallamine (M2 antagonist; final concentration 10^−6^ M), pirenzepine (M1 antagonist; final concentration 10^−6^ M), 1,1-dimethyl-4-diphenylacetoxypiperidinium iodide (4-DAMP) (M3 antagonist; final concentration 10^−8^ M). The concentrations used were comparable with the values of inhibition constant (Ki) reported for rat OLs 5, 6.

### RNA extraction and Real Time-PCR analysis

Total RNA was extracted from U87MG and U251MG tumour cell lines and primary glioma cells using TriReagent (Sigma-Aldrich) supplemented with 250 μg/ml glycogen (Sigma-Aldrich), precipitated with ethanol 70% (Fluka, St. Louis, MO, USA), and digested with DNAse I (Ambion, Foster City, CA, USA).

The expression of the M2 muscarinic receptor subtype was evaluated both by RT-PCR and real time-PCR analysis using the selective primers reported as follows:

***m2:** forward 5′-*CTCCAGCCATTCTCTTCTGG-3′; *reverse 5′-*GCAACAGGCTCCTTCTTGTC-3′;

***18S:** forward 5′-*CCAGTAAGTGCGGGTCATAAGC -3′; *reverse 5′-*AACGATCCAATCGGTAGTAGCG -3′.

For each RNA sample, 1–2 μg of total RNA was reverse transcribed for 60 min. at 37°C, with random hexamers as primers and M-MLV Reverse Trancriptase (Promega, Madison, WI, USA).

A quantity of 50 ng of each cDNA was used as template in each tube for real time-PCR assay. SyBRGreen Jump Start Taq Ready Mix (Sigma-Aldrich) and primers (final concentration 300 nM) or 18s primers were also added at the respective reaction tubes and analysed by I Cycler IQ™ Multicolor Real Time Detection System (Biorad, Hercules, CA, USA). All samples were run in triplicate. The real time-PCR conditions included a denaturing step at 95°C for 3 min. followed by 40 cycles at 95°C for 30 sec.; 60°C for 30 sec. and 75°C for 45 sec. Two cycles were included as final steps: one at 95°C (1 min.) and the other at the annealing temperature specific for each couple of primers used (1 min.). Quantification was performed using a comparative C_T_ method (C_T_ = threshold cycle value). Briefly, C_T_ value was calculated as the mean of the C_T_ each sample performed in triplicate. The differences between the mean C_T_ value of each sample and the C_T_ value of the housekeeping gene (18s) were calculated: ΔC_Tsample_ = C_Tsample_ − C_T18s_. Final results were determined as 2^−ΔΔCT^ where ΔΔC_T_ = ΔCTsample − ΔCTcalibrator.

### Cell viability

The two different glial tumour cell lines, U87MG and U251MG and primary cell cultures obtained from biopsies, were plated onto 96 multiwells and maintained in complete DMEM as described above, and supplemented with 10% or 0.5% FCS. The day after plating, the cells except control samples, were incubated in the presence of the M2 selective agonist arecaidine (final concentration: 10 or 100 μM) for 24, 48, 72 and 96 hrs 25. Cell viability for the arecaidine treated cells was assessed by 3-(4,5-dymethyl thiazol 2-y1)-2,5-diphenyl tetrazolium bromide (MTT, Sigma-Aldrich, St. Louis, MO, USA), according to Mosman's procedure 32. After MTT addition, cultures were incubated for 4 hrs. The medium was then removed and 100 μl of DMSO was added to each well to solubilize the dark blue crystals. The plates were then read at wavelength of 570 nm. Under the same experimental conditions we evaluated the presence of dead cells by trypan blue staining (1:10 v/v).

### Western immunoblotting

To detect the expression of M2 and TP53, protein extracts were fractionated by 10% SDS-polyacrilamide gel (PAGE) electrophoresis and transferred to PVDF sheets (Millipore, Billerica, MA, USA). Membranes were incubated in 5% of non-fat dry milk in PBST (phosphate buffer saline + tween 20) and then incubated with monoclonal anti-M2 (1:500; Abcam ab2805) and monoclonal anti- p53 antibodies (1:10000; Sigma-Aldrich) overnight at 4°C. Blots were then washed and incubated with secondary antibodies conjugated to horseradish-peroxidase (Promega). The reaction was revealed by ECL chemiluminescence reagent [Euroclone, Pero (MI), Italy].

To analyse the effects of arecaidine on PI3K activity, U251 and U87 cells, at 1 day of sub-culturing, were serum starved for 24 hrs, incubated for 30 min. with 0.1% DMSO or with 10 μM LY294002 as inhibitor of PI3K activity (Merck Biosciences, Billerica, MA, USA), and stimulated with 100 μM arecaidine. The levels of Akt, phosphorylated at serine 473 and of p44/42 MAPK, phosphorylated at threonine 202/tyrosine 204, were detected by using the anti-phospho-Akt (Ser473) and anti-phospho-p44/42 MAPK (Thr202/Tyr204) antibodies, and compared with the expression of total p44/42 MAPK, detected with an anti-p44/42 MAPK antibody (Cell Signaling Technology, Danvers, MA, USA).

### Immunocyto- histochemistry analysis

U87MG and U251MG cell lines were plated onto 35-mm diameter dishes in complete DMEM containing 10% FCS. Then, the cells were washed twice with PBS pH 7.4 and fixed for 20 min. in 4% paraformaldehyde in PBS, at room temperature. The cells were washed three times in PBS and incubated for 45 min. in PBS 1X pH 7.4 containing 10% normal goat serum (NGS), and 1% bovine serum albumin (BSA, Sigma-Aldrich, St. Louis, MO, USA). The excess of the blocking solution was removed and the cells were then incubated overnight at 4°C with the primary antibody (mouse anti M2, Abcam), diluted 1:200 in PBS containing 1% NGS, 1% BSA. The next day, the cells were washed twice (10 min. each) in PBS plus 1% BSA and then incubated for 1 hr at room temperature with anti mouse-Alexa 594 conjugated antibody (Promega, Madison, WI, USA) diluted 1:250 in PBS plus 1% BSA. After two washes in PBS plus 1% BSA the cultures were mounted with glycerol/PBS (3:1, v/v). Negative controls were obtained by omitting the primary antibody.

Immunohistochemical analysis was performed on paraffin-embedded surgical specimens. Tumour grade were established according to the WHO classification. Paraffin sections were de-paraffined and heat-induced epitope retrieval was carried out by incubation in TEG buffer solution (10 mM Tris and 0.5 mM EGTA). After blocking endogenous peroxidase activity by incubation in 1.5% hydrogen peroxide (H_2_O_2_), the sections were incubated overnight at 4°C with antibodies against the M2 receptor (1:200; Abcam ab2805); Ki67(1:200, Dako, Glostrup, Denmark) and p53 (1:500; Sigma-Aldrich). The next day the peroxidase-conjugated secondary antibodies were incubated for 1 hr and then detected with an ABC Universal Quick kit (Vector Laboratories, Burlingame, CA, USA) following the manufacturer's procedure. The sections were counterstained with haematoxylin-eosin. Samples of distinct areas of each tumour and serial sections of the same area were included in the analysis.

### Flow cytometry analysis

The cells were plated onto 90-mm diameter dishes at a density of 5 × 10^5^ cells/dish. The day after plating, the cells, excluding control samples, were treated with 10^−4^ M arecaidine for 24, 48, 72 and 96 hrs. At the end of the treatment, cells were incubated for 20 min. with 45 μM bromodeoxyuridine (final concentration) (BrdUrd, Sigma-Aldrich), collected by trypsinization, centrifuged for 10 min. at 1,000 r.p.m. and then fixed in methanol/PBS 1:1 (v/v).

To identify cells in S phase, DNA content and BrdUrd incorporation were determined in simultaneous analysis by staining with propidium iodide (PI) and anti-BrdU respectively.

Partial DNA denaturation was performed by incubating the cells in 3N HCl for 45 min., followed by neutralization with 0.1 M sodium tetraborate. Samples were then incubated with monoclonal anti-BrdUrd (1:50 v/v; Dako) for a further 30 min. at room temperature, washed twice with 0.5% Tween-20 in PBS and incubated for 30 min. with antimouse Alexa fluor-conjugated (1:600). Samples were washed twice with PBS and finally stained with 10 μg/ml PI for 15 min. at room temperature. Flow cytometry analysis was performed with a flow cytometer Coulter Epics XL with 488 nm wavelength excitation and 10^4^ events were collected for each sample. Monoparametric (DNA histograms) and biparametric (DNA content *versus* BrdUrd content) analysis were performed using WinMDI 2.7 software.

### Apoptotic cell detection

Apoptotic cells were quantified by flow cytometry analysis by propidium iodide (PI) staining. Briefly, 2 × 10^6^ cells were suspended in 2 ml of PBS buffer containing 0.1% Triton X-100 (Sigma-Aldrich) and incubated for 5 min. at room temperature. Cells were subsequently stained with 10 μg/ml PI and analysed using a Coulter Epics XL flow cytometer. For each sample, 10,000 events were recorded. Cells with a hypodiploid DNA content and a higher granulosity (SSC) than that of G0-G1cells were quantified as apoptotic cells 33, 34.

Apoptotic cells were also evaluated by ELISA detection of cytoplasmic nucleosomes kit (Roche, Basel, Switzerland). Determination of cytoplasmic histone-associated DNA fragments was performed following the manufacturer's instructions. The results are expressed as percentage of optical density, resulting from the activity of peroxidase-conjugated anti-DNA antibody complexed with cytoplasmic nucleosomes of treated cells, compared with the controls.

### Kinetic analysis of arecaidine and temozolomide chemosensitivity

A chemosensitivity test was performed for arecaidine and temozolomide using concentrations ranging between 12.5–100 μM and 100–1000 μM respectively. Cells were seeded and grown in 96-well plates at variable numbers, taking into account the growth suppressive effects of the drugs, to ensure that all experiments were performed during the exponential growth phase.

MTT assays were performed to determine the fraction of cells surviving after exposure to the tested agents.

Briefly, the cells were seeded at the density of 2,000 cells/well, after 24 hrs the cells were treated with different drug concentrations for 24, 48 and 72 hrs. The analysis was performed in quadruplicate for each condition in four to five independent experiments.

### M2 siRNA transfection

Four different siRNAs targeting specific sequences of human M2 receptors (CHRM2; ID1129) and a positive control of transfection Chromo-GAPDH siRNA were synthesized by Riboxx Life Sciences (Radebeul, Germany). The sequences of the four CHRM2 siRNAs were as follows:

- (siRNA 1129-1) sense, 5′- AUUUACUACUAAAUCCUCCCCC-3′, antisense 5′-GGGGGAGGAUUUAGUAGUAAAU-3′;
- (siRNA1129-2) sense 5′- AUGUAGCCCAUUUCUUCCCCC-3′, antisense 5′-GGGGGAAGAAAUGGGCUACUA;
- (siRNA 1129-3) sense 5′-UCCUUUGAGUUUCAGGCUGCCCCC-3′, antisense 5′- GGGGGCAGCCUGAAACUCAAAGGA-3′;
- (siRNA 1129-4) sense 5′-AGUUACACCUUGACCUAACCCCC-3′, antisense 5′-GGGGGUUAGGUCAAGGUGUAACU-3′.

The cells were plated in 6-well plates (15 × 10^4^ cells/well) and cultured in 2 ml DMEM cointaining 10%FCS and 1% NEAA until the cells were 70% confluent. The siRNAs were pre-mixed with RiboxxFect according to manufacturer's instructions and then added to wells. The efficiency of the transfection was evaluated by transfecting in separate wells Chromo-GAPDH siRNA. The ability of the siRNA pool to affected CHRM2 expression was tested using three different concentrations of siRNA (10, 20 and 40 nM/well) and then evaluating M2 receptor expression by Western blot analysis 72 hrs after transfection.

### Statistical analysis

t Student's and one-way anova tests followed by Bonferroni's post-test were used to evaluate statistical significance within different samples. The results were considered statistically significant at *P* < 0.05 (*), *P* < 0.01 (**) and *P* < 0.001 (***).

## Results

### M2 receptors in glioblastoma cells

**Fig. 1:**
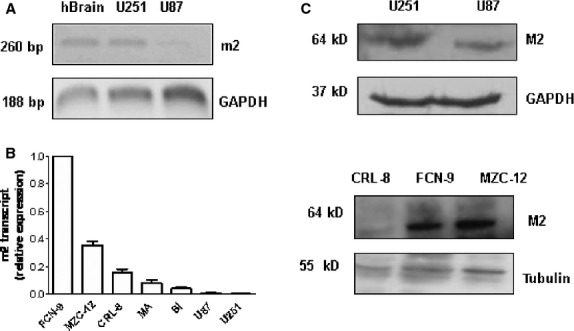
M2 expression in glioma cells. (**A**) RT-PCR analysis of m2 transcript espression in two glioblastoma cell lines (U251 and U87). RNA from human brain was used as positive control, the GAPDH was used as housekeeping gene. (**B**) Levels of expression of m2 transcript by real time PCR in five primary cell cultures obtained from biopsies and in glioma cell lines U87 and U251. The levels of the transcript were normalized with the housekeeping gene (18s) and compared to the reference sample FCN-9. (**C**) Expression of M2 protein by Western blot analysis in both cell lines (U251 and U87) and in three primary cell cultures (CRL-8; FCN-9; MZC-12). GAPDH and tubulin were used to normalize the intensity of the bands.

**Fig. 2:**
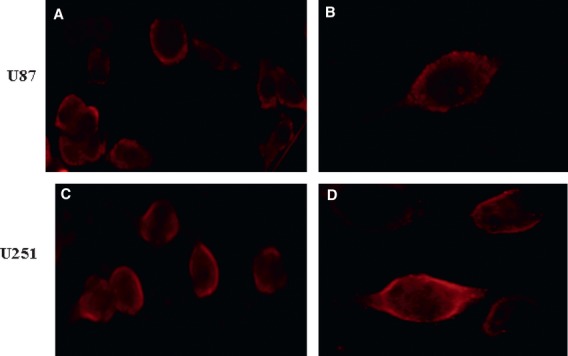
Immunocytochemistry analysis of M2 receptor in U87 (**A** and **B**) and U251 (**C** and **D**) cell lines. **A** and **C** (×150); **B** and **D** (×300). Negative control obtained omitting primary antibody did not show any staining (data not shown).

The expression of M2 receptors was investigated in glioblastoma cell cultures and in human fresh glioma specimens. The RT-PCR analysis showed that all stable cell lines (U251MG and U87MG) express the M2 transcript; however, M2 expression in U87MG appeared lower than that in U251MG (Fig. 1A). The real time-PCR analysis showed also that M2 mRNA levels were significantly higher in primary cultures as compared to stable cell lines (Fig. 1B). Western blot analysis confirmed the high expression of M2 receptor in U251MG (Fig. 1C upper panel). Similar results were obtained by immunocytochemistry. Although immunocytochemistry is not a quantitative technique, the intensity of the M2 staining was more evident in U251 (Fig. 2B and D) than in U87 cells (Fig. 2A and C). The amount of M2 receptors revealed in primary cell cultures by Western blot analysis appears in agreement with real time-PCR data (Fig. 1B lower panel).

**Fig. 3:**
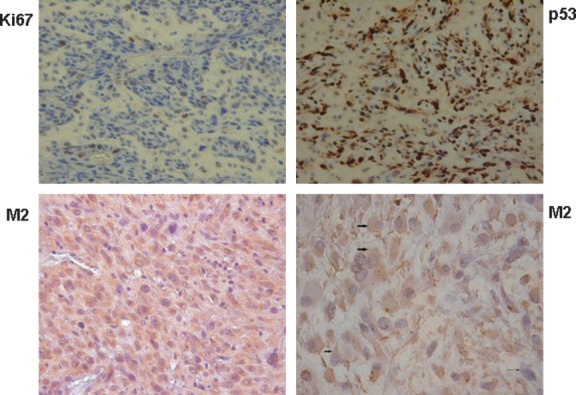
Immunohistochemistry of M2 receptors in fresh glioma biopsy. The paraffin-embedded sections were used to analyse the expression of Ki67, p53 and M2 receptor expression. M2 receptors appear localized in the tumour central area and in giant-multinucleated cells. Rare positive cells were located along the vascular basal lamina and perivascular region (100×). At higher magnification, short arrows indicate positive glioblastoma cells and the long arrow indicates negative endothelial-like cell (200×).

Finally, we investigated the expression and localization of the M2 muscarinic receptor in fresh biopsies, from glioblastoma grade IV (*n* = 6). A diffuse M2 staining was observed in the cytoplasm and membranes in all analysed tumours (Fig. 3). Most positive cells were identified as diffusely infiltrating the tumour central area. Rare positive cells were located along the vascular basal lamina and perivascular region. A strong positive staining was also present in giant-multinucleated cells. A representative sample is shown in Figure 3. Ki67 and p53 immunostaining, respectively, identifying proliferating cells and the presence of mutated protein is also reported (Fig. 3).

### Analysis of cell growth

Previous data have indicated that the selective activation of M2 receptors by the agonist arecaidine caused a decrease in cell proliferation in glioblastoma cell lines in a time and dose dependent manner 28. Here we extend this observation to several primary cell cultures derived from tumour biopsies in addition to the U251MG and U87MG. These two cell lines express different mutations and they can be considered as representative of the genetic settings widely represented among glioma tumours. The sensitivity to arecaidine in primary cell cultures was analysed by MTT assay. We used five different primary cell cultures characterized by different mutations: MZC and CRL-8 are p53 mutated, MTR presents a copy of deleted p16, FCN-9 shows a frame mutation in the p53 gene, while BML bears wild-type p53 and p16 genes 35. The MTT assay was performed in presence of high (10%) or low FCS concentration (0.5%).

**Fig. 4:**
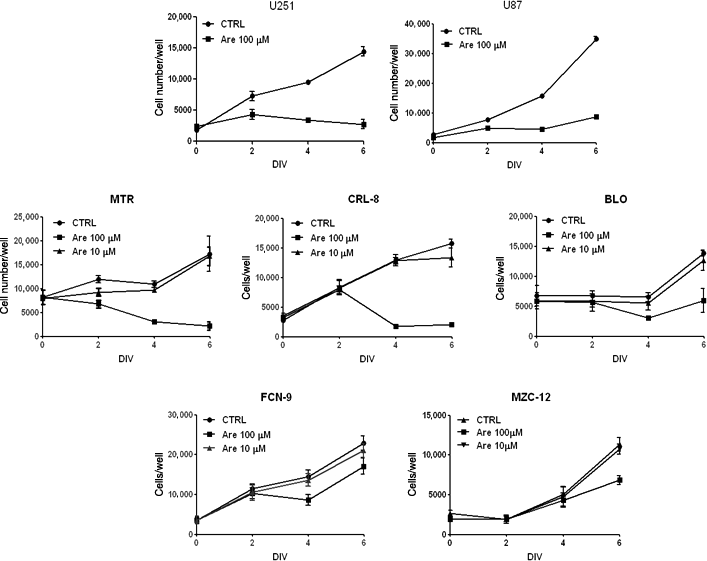
Effect of arecaidine on primary cell lines obtained from biopsies. Time and dose dependent analysis of cell growth by different doses of arecaidine (10–100 μM) has been evaluated in five different primary cell cultures (MTZ, CRL-8, BLO, FCN-9, MZC-12). Data are the mean ± SEM of three independent experiments performed in triplicate. (DIV: day *in vitro*).

All primary cell cultures and stable cell lines showed a significant decrease in cell proliferation in presence of 100 μM arecaidine and 10% FCS (*P* < 0.001, arecaidine *versus* control; Fig. 4). The most sensitive were CRL-8, MTR, BML and U251 cell lines indicating that the efficacy of arecaidine is independent of gene status. Comparable results were obtained in presence of low concentrations of FCS (data not shown).

Human fibroblasts from normal tissue were also tested for their sensitivity to arecaidine, demonstrating that the M2 agonist caused a reduction in cell proliferation but not in cell survival (data not shown).

To compare the cytotoxic effects of arecaidine and temozolomide (TMZ), we evaluated the IC_50_ values in primary cell cultures and in U87MG and U251MG lines. Table 1 shows that arecaidine IC_50_ was significantly lower than TMZ IC_50_ (*P* < 0.001). In addition, the IC_50_ value range for arecaidine in different cell lines was less extensive as compared to TMZ IC_50_; in fact TMZ IC_50_ for CRL-8 was 10 times lower than for MZC-12 (Table 1).

**Table 1:** Values of IC_50_ calculated for temozolomide and arecaidine in U87 and U251 stable cell lines and in three primary cell lines obtained from biopsies (FCN-9, MZC-12, CRL-8)

Cell lines	TMZ IC_50 μM_	SD	Arec IC_50 μM_	SD
FCN-9	283.5	13.7	121.3	8.5
MZC-12	1049.0	31.6	123.0	15.6
CRL- 8	94.3	24.2	58.5	1.3
U87	864.1	40.9	64.4	1.1
U251	404.5	19.5	65.8	0.7

**Table 2:** Percentage of cells in G1, S, G2/M phases after 100 μM arecaidine treatment. The data are the mean ± SEM. *P* values are also reported (arecaidine treated cells *versus* control)

U251	%G1	±SEM	*P*	%S	±SEM	*P*	%G2/M	±SEM	*P*
Control	39.39	±4.57		43.49	±3.59		17.12	±1.46	
Arecaidine 100 μM 24 hrs	29.16	±2.29	0.074	39.87	±3.09	0.3600	30.97	±2.91	0.0017
Arecaidine 100 μM 48 hrs	44.09	±2.23	0.610	19.97	±2.43	0.0005	35.94	±3.48	0.0007
Arecaidine 100 μM 72 hrs	44.68	±7.23	0.532	13.80	±2.80	0.0001	41.52	±8.88	0.0150
Arecaidine 100 μM 96 hrs	49.97	±8.67	0.263	14.77	±3.03	0.0012	35.26	±8.78	0.0270

### M2 receptor activation inhibits cell cycle progression

**Fig. 5:**
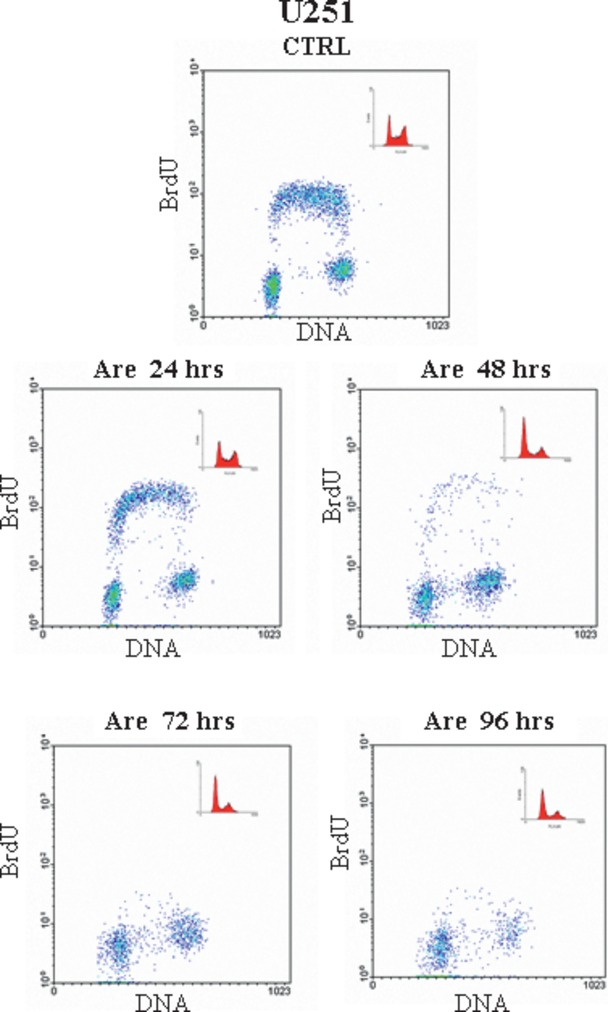
Bivariate analysis of BrdUrd incorporation (ordinate) and DNA content (abscissa) in U251 cells at 24, 48, 72 and 96 hrs after 100 μM arecaidine (Are) treatment. The BrdUrd labelled cell fraction appears dramatically reduced in arecaidine treated cells.

**Fig. 6:**
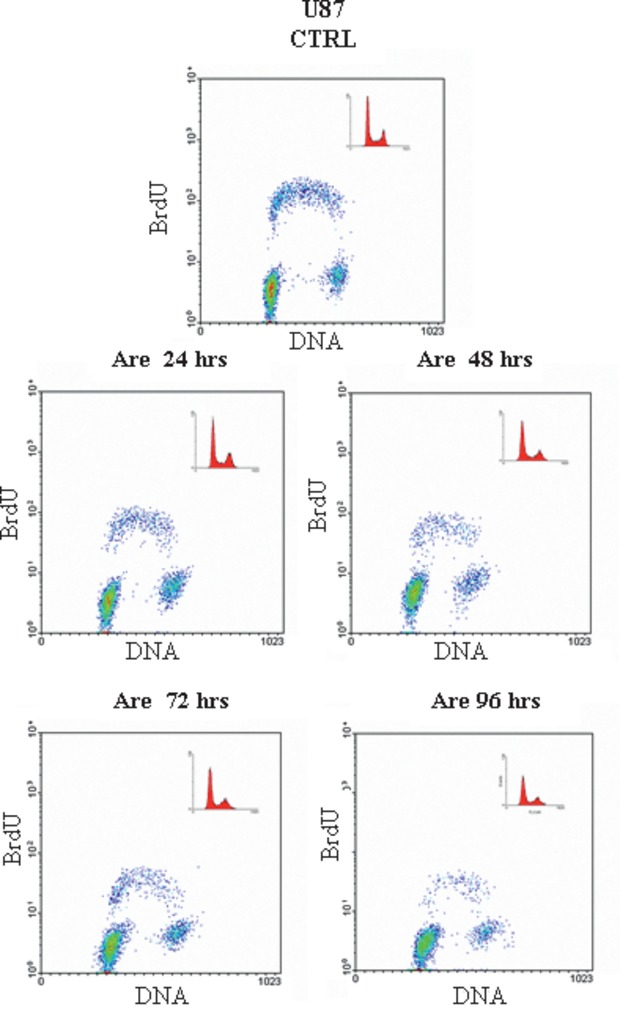
Bivariate analysis of BrdUrd incorporation (ordinate) and DNA content (abscissa) in U87 cells at 24, 48, 72 and 96 hrs after 100 μM arecaidine (Are) treatment. The BrdUrd labelled cell fraction appears reduced in arecaidine treated cells.

To confirm the arecaidine cell proliferation inhibition, we performed cell cycle analysis on U251MG cell lines maintained in the presence of 100 μM arecaidine for 24, 48, 72 and 96 hrs. Before harvesting, cell cultures were pulsed with BrdUrd for 20 min. to monitor S phase progression. The bi-parametric analysis of BrdUrd labelling *versus* DNA content, measured by propidium iodide, allowed the analysis of cell progression through the G1/S/G2 phases and a more obvious identification of cells in S phase. As shown in Figure 5 and Table 2, the U251 cells showed a dramatic decrease in the BrdUrd labelled cell fraction as early as 24 hrs after arecaidine treatment. A progressive decrease in the percentage of cells in the S phase after prolonged treatment was also observed with a progressive accumulation of the cells in the G2/M phase, evident after 48 hrs. Arecaidine was also able to inhibit cell proliferation in the U87 cells (Fig. 6), where we observed a decrease in the percentage of cells in S phase at all treatment times. Progressive accumulation of cells in the G1/S phase (Fig. 6 and Table 3), suggests that arecaidine causes an arrest of cell cycle progression in the G1/S transition.

**Table 3:** Percentage of cells in G1, S, G2/M phases after 100 μM arecaidine treatment. The data are the mean ± SEM. *P* values are also reported (arecaidine treated cells *versus* control)

U87	%G1	±SEM	*P*	%S	±SEM	*P*	%G2/M	±SEM	*P*
Control	54.14	±1.41		30.59	±0.98		15.09	±0.47	
Arecaidine 100 μM 24 hrs	57.95	±1.55	0.1500	23.87	±1.45	0.0142	18.09	±1.00	0.0392
Arecaidine 100 μM 48 hrs	62.96	±1.42	0.0043	16.76	±1.46	0.0001	19.85	±1.62	0.0361
Arecaidine 100 μM 72 hrs	62.84	±2.07	0.0015	17.51	±1.23	0.0001	19.52	±1.60	0.0456
Arecaidine 100 μM 96 hrs	63.97	±1.04	0.0051	15.19	±1.97	0.0005	20.44	±0.61	0.0010

**Fig. 7:**
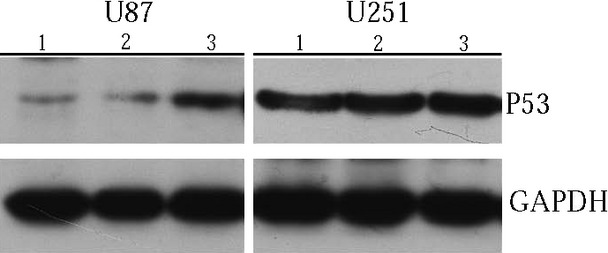
Immunoblotting analysis showing p53 protein expression in U87 and U251 cells. Lane 1: protein extracts from untreated cells (control); lane 2: protein extracts by 50 μM arecaidine-treated cells; lane 3: protein extracts by 100 μM arecaidine treated cells. GAPDH was used to normalize the amount of total proteins loaded in each lane.

To explain the different behaviours of two established cell lines, we investigated the effect of arecaidine treatment on p53 expression. In the U87MG cells, which express the wild-type p53, the arecaidine treatment induced a dose dependent upregulation of p53 expression (Fig. 7). This effect may be responsible for the arrest of the cell cycle in the G1/S phase. Conversely, in the U251 cells, where p53 is mutated, the expression of the protein is not affected by arecaidine treatment (Fig. 7). We hypothesize that the accumulation of U251 cells in the G2/M transition, might be dependent on the inability of the mutated p53 to arrest cell proliferation at the G1/S checkpoint (Fig. 7).

### M2 receptor activation affects cell survival inducing apoptosis

**Fig. 8:**
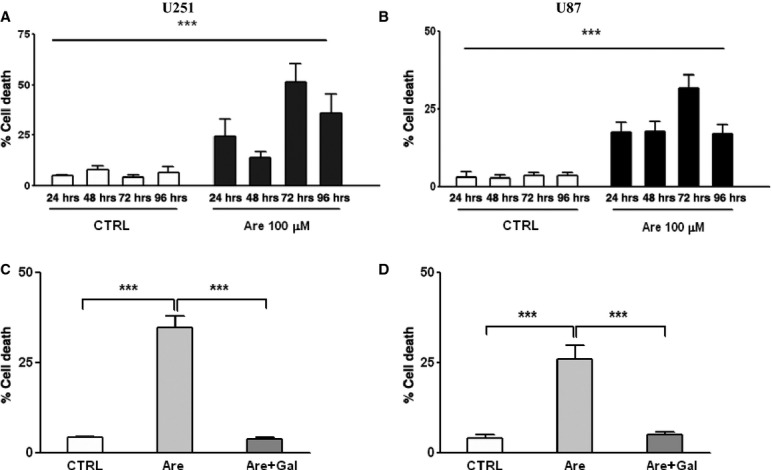
Cell death evaluated by trypan blue staining. The number of the dead cells present in untreated cells and in arecaidine treated cells was calculated as the percentage of dead cells/total cells (upper panels). The trypan blue staining was also used to evaluate the ability of M2 antagonist gallamine (10^−6^ M) to counteract the arecaidine effect on cell survival (lower panels). Data represent the mean ± SEM of three independent experiments performed in triplicate (***P < 0.001).

To assess whether the decrease in cell number, observed after arecaidine treatment (Fig. 4) was only dependent on a lower cell proliferation or apoptosis induced by arecaidine, we evaluated the appearance of dead cells following arecaidine treatment using several approaches. As reported in Figure 8, trypan blue staining of U251MG and U87MG cells following arecaidine treatment at two different concentrations (10–100 μM) for 24, 48, 72 and 96 hrs, showed that in both cell lines arecaidine induced cell death already after 24 hrs of treatment. The toxic effect of arecaidine was more evident at 100 μM (for 10 μM, data not shown); in fact under this condition the number of dead cells increased with time, reaching a maximum of 25% in U87 cells (96 hrs), and of 50% in U251 (96 hrs; Fig. 8A and B). The ability of arecaidine to induce cell death was reverted when the cells were co-treated for 72 hrs in presence of the M2 antagonist gallamine (10^−6^ M), confirming that the arecaidine effects described above were dependent on M2 receptor activation (Fig. 8C and D).

**Fig. 9:**
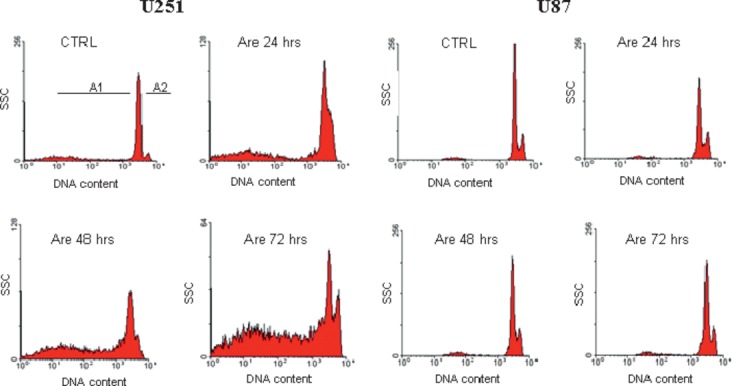
Cytometric analysis of the apoptosis in control and arecaidine treated-glioma cell lines (final concentration 100 μM; see also Table 4; A1, apoptotic cells; A2 viable cycling cells).

**Fig. 10:**
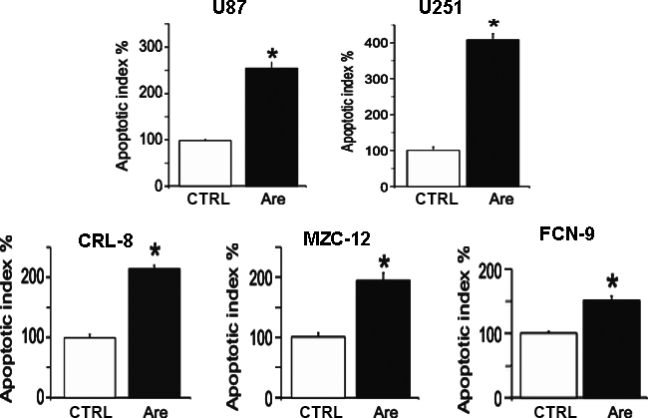
Cell death induced by arecaidine was tested using an ELISA assay aimed at quantifying cytoplasmic nucleosomes (*P < 0.05 are *versus* control).

To assess whether or not arecaidine induced apoptotic cell death, we followed two different approaches. First we determined by FACS analysis the fraction of cells with hypodiploid DNA content and higher granulosity (SSC). This fraction was assumed to be apoptotic cells. In Figure 9 we show that arecaidine increased the percentage of U87MG and U251MG hypodiploid cells. U251 cells appeared much more sensitive to the treatment, in fact after 72 hrs of treatment 60% of cells with apoptotic features were found in the U251MG cell lines, whereas in the U87MG cell line, the percentage of apoptotic cells was 14.52% (see Table 4). These data were confirmed by quantifying cytoplasmic nucleosomes, considered to be a hallmark of apoptosis, with an ELISA assay. As shown in Figure 10, treatment with 100 μM arecaidine for 72 hrs induced an increase in the percentage of cytoplasmic nucleosomes both in cell lines (U87 and U251) and in primary cell cultures (CRL-8, MZC-12, FCN-9). The ability of arecaidine to induce apoptosis appears to be higher in glioblastoma cell lines than in primary cultures; in particular U251MG cells displaying the highest sensitivity to apoptosis were arecaidine-induced cells. These data appear in agreement with FACS analysis data.

**Table 4:** Percentage of the apoptotic cells present in U87 and U251 cultures after 100 μM arecaidine treatment. The data reported are the mean ± SEM. *P* values are also reported (arecaidine treated cells *versus* control)

	U87% apoptotic cells ± SEM	*P*	U251% apoptotic cells ± SEM	*P*
Control	7.41 ± 1.04		16.16 ± 4.50	
Arecaidine 24 hrs	17.56 ± 3.05	0.008	27.84 ± 3.74	0.130
Arecaidine 48 hrs	14.79 ± 1.99	0.010	37.33 ± 7.40	0.050
Arecaidine 72 hrs	14.52 ± 3.99	0.120	60.51 ± 6.55	0.001

### Arecaidine effects on PI3K/Akt pathway

**Fig. 11:**
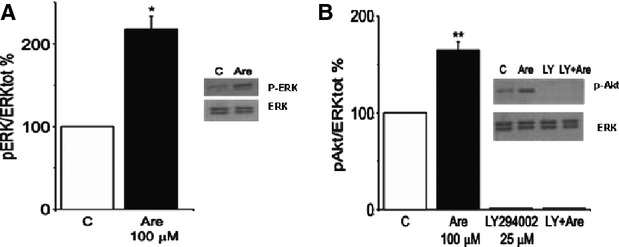
Western blot analysis for pERK (**A**) and pAkt (**B**) in U251 cells. The total ERK was used to normalize the intensity of the bands. The inhibitor of PI3K, LY294002 (25 μM) was used to confirm the involvement of PI3K in pAkt activation (*P < 0.05; **P < 0.01; 100 μM arecaidine *versus* control).

To further investigate the mechanism leading to the decrease in cell proliferation and survival caused by arecaidine, we have analysed the ability of the M2 agonist to modulate PI3K/Akt and ERK pathways in two glioblastoma cell lines. Our results confirm that both cell lines constitutively express the phosphorylated form of Akt in the absence of serum, a characteristic which is typical of PTEN-deficient glioblastoma cells (Fig. 11). Moreover, we have observed that arecaidine up-regulated p-Akt in U251 cells; the presence of LY294002, an inhibitor of PI3K, inhibited the expression of p-AKT both in treated and in untreated cells, suggesting that the p-AKT production was dependent on PI3K pathway activation (Fig. 11B). At the same time, arecaidine induced an upregulation also of p- ERK in U251 cells (Fig. 11A). Conversely the M2 agonist did not have any effect on p-AKT and p-ERK expression in the U87 cells (data not shown).

### Effects produced by muscarinic receptor antagonists and M2-silencing

Although the competition experiments with M2 antagonist gallamine have suggested the direct involvement of M2 receptor in the inhibition of glioma cell proliferation and in the induction of apoptosis (subsection 4 and Ref. 28), to exclude that the arecaidine treatment may involve other muscarinic receptor subtypes, we have performed two different experiments: (i) analysis of cell growth in the presence of M1 and M3 antagonists; (ii) M2 receptor silencing.

**Fig. 12:**
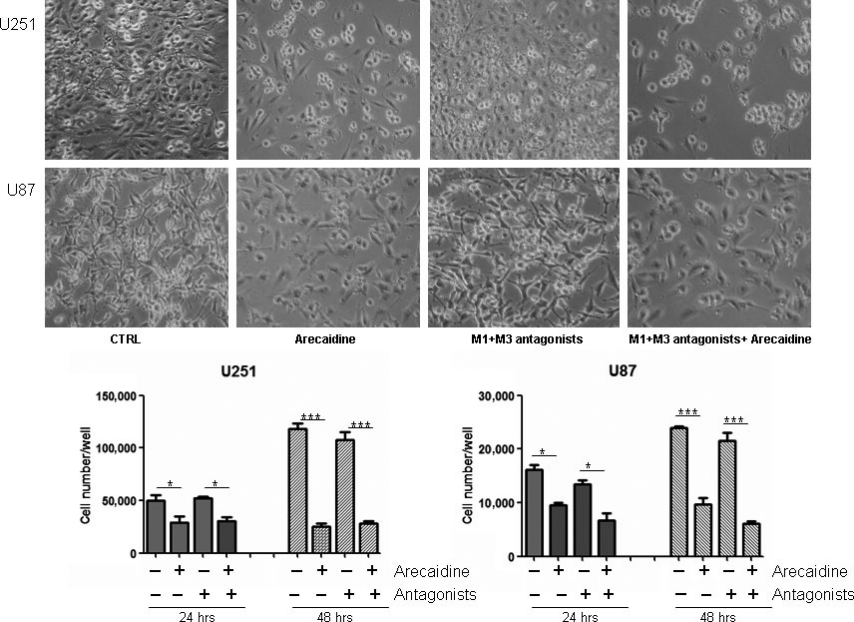
Effects of M1 and M3 muscarinic receptor antagonists on glioma cell growth. The photographs (upper panels) indicate the U251 and U87 cell lines cultured in absence of muscarinic agonists and antagonists (ctrl), maintained for 24 hrs in presence either of M2 agonist arecaidine (100 μM), or of M1 antagonist pirenzepine (10^−6^ M) plus M3 antagonist 4-DAMP (10^−8^ M) alone, or of arecaidine in co-presence with M1 and M3 antagonists (100×). The botton panel reports the data obtained by MTT analysis performed on both glioma cell lines maintained in the same experimental conditions for 24 and 48 hrs. The values are the mean ± SEM of two independent experiments performed in triplicate. The values were considered significant *P* < 0.05 (*); *P* < 0.001 (***).

**Fig. 13:**
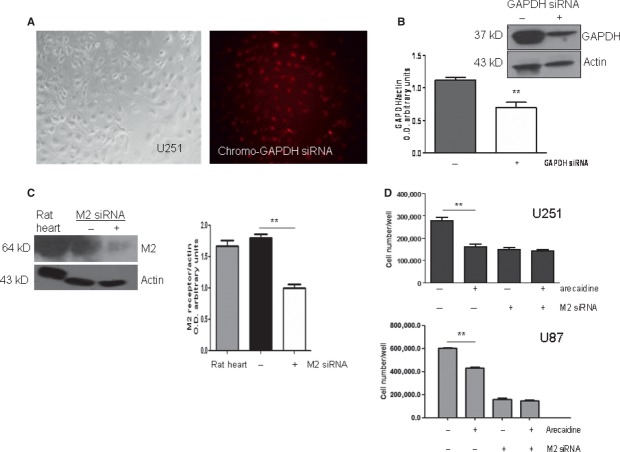
Effect of M2-silencing on U251 and U87 cell growth. (**A**) U251 transfected with Chromo GAPDH siRNA for 72 hrs (red) used as control of transfection (100×), (**B**) Western blot and densitometric analysis of the GAPDH espression in U251 cells in absence and in presence of Chromo-GAPDH siRNA (after 72 hrs of transfection); actin was used to normalize the bands (**C**) Western blot and densitometric analysis of the M2 receptor espression in U251 cells in absence and in presence of a pool of M2-siRNA (after 72 hrs of transfection); rat heart was used as control of M2 expression; actin was used to normalize the bands. (**D**) MTT analysis performed in two glioma cell lines after 72 hrs of siRNA transfection and 24 hrs of arecaidine treatment. The values are the mean ± SEM of two independent experiments performed in triplicate. The values were considered significant *P* < 0.01 (**).

To exclude the ability of arecaidine to activate also other muscarinic receptor subtypes we have cultured U251MG and U87MG lines in 24 well trays for 24 and 48 hrs, in presence of either the M1 antagonist pirenzepine (final concentration 10^−6^ M) or M3 antagonist 4-DAMP (final concentration 10^−8^ M) alone or in addition to arecaidine (final concentration 100 μM). The MTT assay was used to evaluate the cell number in the different experimental conditions. As reported in Figure 12 the treatment with M1 and M3 antagonists did not affect cell growth in both glioma cell lines, as indicated by a comparable cell number in control conditions and in antagonists treated cells, at 24 and 48 hrs of treatment. Arecaidine, as previously reported 28 inhibits cell growth in both glioma cell lines and this effect was not counteracted by the presence of M1 and M3 antagonists. Finally, to confirm that the effect mediated by arecaidine was completely dependent on M2 receptor activation and to exclude a cytotoxic effect of the molecule, we have treated glioma cell lines with arecaidine after M2 receptor silencing using a mix of four siRNAs targeting human M2 receptor. Seventy per cent efficiency was obtained in siRNA transfection experiments, as indicated by the inhibition of GAPDH expression, following transfection of Chromo-siRNA GAPDH (20 nM/well) used as positive control (Fig. 13A and B). The use of a mix of four siRNAs (40 nM each/well) specific for CHRM2 reduced by 30–40% M2 expression in U251 cells, as indicated by Western blot analysis performed 72 hrs after transfection (Fig. 13C). Although the transfection caused a decrease in cell number possibly due to lipofectamine toxicity, 24 hrs of treatment with arecaidine after M2 silencing showed the inability of M2 agonist to inhibit cell proliferation in both glioma cell lines (Fig. 13D).

## Discussion

In recent years, acetylcholine and muscarinic receptors as possible tools in cancer therapies have been considered 1, 12. Muscarinic receptors appear to be involved in brain tumours. It has been shown that ACh stimulates cell proliferation in low-grade astrocytomas through M3 subtype activation 26. Glioma cell lines have been shown to respond to cholinergic stimulation; in fact the selective stimulation of muscarinic receptors by muscarine inhibits cell migration *via* opening Ca++ dependent K+ channels. This effect appears counteracted by 4-DAMP, indicating that it is mediated by M3 subtype 36. In fact the M3 subtype has been shown to be the subtype mainly involved as reported for the effects on growth and propagation of different tumours, including brain tumours 12.

Recently we observed that in glioblastoma cell lines the use of the non-selective agonist muscarine did not modify cell growth, but that the selective activation of M2 receptors caused a significant decrease in cell proliferation 28. These results prompted us to further investigate the role of M2 receptors in glioma cells.

First of all, we have described the expression of M2 receptors in the glioblastoma cell lines U87MG e U251MG, comparing their level of expression with levels found in primary cell cultures obtained from fresh glioma biopsies. We found that M2 receptors are expressed both as transcripts and as protein with different levels among the cell lines. In fact, the M2 protein is abundantly present in stable cell lines in particularly in U251MG whereas it is differentially expressed in the primary cultures. We also confirmed the presence of M2 receptors in sections from glioma biopsies.

Our studies of cell proliferation performed by MTT assays have clearly indicated that the M2 agonist arecaidine is able to decrease cell proliferation in all analysed glioma cell lines, although they carry different mutations (see also Ref. 28). However, arecaidine responsiveness varies among different cell lines; this may, at least in part, be dependent on the levels of M2 receptor expression. Considering that the arecaidine effects are time and dose dependent 28, all the subsequent experiments were performed at the concentration of 100 μM, the dose that caused the more pronounced decrease in cell growth. The analysis of the cell cycle by FACS has shown that the arecaidine treatment induced an arrest of the cell cycle in the two stable cell lines, although some differences have been observed. In fact the M2 agonist caused the arrest of cell cycle in G1/S for U87MG cells and in G2/M for U251MG with a significant decrease in the percentage of cells in the S phase in both cell lines. These differences may depend on the different mutations present in these cells. Supporting this hypothesis is the observation that the M2 agonist arecaidine modulates the expression of p53 in the U87MG but not in the U251MG cell line, which has mutated copies of the p53 gene, causing the abundant protein expression and accumulation. On the other hand, in the U87MG cells, which have wild-type copies of p53 and where the tumour suppressor gene PTEN is deleted, the expression of p53 is very low, but is progressively up-regulated following arecaidine stimulation. Therefore, there could be a link between p53 activation and cell cycle alterations in this cell line. Another important consequence of M2 activation in glioblastoma cells is the time dependent decrease in cell survival. The involvement of M2 receptors in mediating these effects was confirmed by the use of the M2 antagonist gallamine. In fact co-stimulation with both agonist and antagonist results in the inhibition of arecaidine-induced cell death in addition to inhibition of cell proliferation as previously demonstrated 28. On the basis of these data we can exclude the involvement of other muscarinic receptors in mediating these effects, considering that the use of M1 and M3 antagonists do not counteract the arecaidine effect on cell proliferation. On the other hand low espression levels of M2 receptors, after specific siRNAs transfection, abolish arecaidine effects. Altogether these data support the hypothesis that arecaidine effect is dependent on M2 receptor activation.

Moreover, we also demonstrated that arecaidine can induce the apoptotic cell death in both stable and primary cell lines, as shown by the presence of cytoplasmic nucleosomes and cellular fragments with hypodiploid DNA content, detected by ELISA and FACS assays respectively. The percentages of apoptotic cells, demonstrated by the two assays were comparable. As both tumour cell proliferation and resistance to apoptosis are mediated by PI3K/AKT in glioma and other tumour types 37–39, we have also tested the ability of arecaidine to counteract this pathway. Surprisingly we found that M2 activation up-regulates pAkt and that the PI3K inhibitor LY294002 can revert pAkt activation. Moreover, we have also observed that ERK activity, generally considered to be involved in the resistance to apoptosis 40 and demonstrated to mediate apoptotic induction in glioblastoma cells 41, 42, was activated by arecaidine stimulation, confirming a possible involvement in arecaidine induced apoptotic cell death. Instead these data allow excluding the fact that cell arrest and apoptosis were dependent on the downregulation of PI3K/Akt signalling. Although the abnormal activation of AKT has been thought to play a major role in resistance to apoptosis and chemotherapy in a large percentage of human glioblastomas 37, it has been proposed recently that under certain conditions the abnormal activation of the PI3K/AKT pathway in PTEN-deficient glioblastoma cells could represent a disadvantage for cell survival 43. This may explain, at least in part, why arecaidine induced cell death in U251MG cells was stronger than that in U87MG cells.

The chemotherapy approach for patients diagnosed with GBM has been considered controversial. In fact treatment with a single agent frequently shows limited benefits. TMZ is the *gold standard* in glioma therapeutic protocols. Although the use of this drug causes an improvement in medium term survival, it is ineffective in resistant cases of primary glioma 27. In this study, we have compared the sensitivity of cell lines to the conventional drug TMZ with that for the M2 agonist arecaidine. Both drugs are effective, with arecaidine IC50 values significantly lower than those for TMZ (*P* < 0.001). Moreover, arecaidine seems to induce inhibition of cell growth more efficiently than TMZ, at least in stable cell lines. Recent publications have focused their attention on the possible role of muscarinic receptors in the control of tumour cell growth 19, 21, 44. Data reported here show for the first time that the M2 receptors can counteract tumour cell proliferation and affect cell survival in glioma cells *in vitro*. Interestingly, the M2 agonist arecaidine shows better result as compared to TMZ, and is effective on different glioma cell cultures, characterized by distinct types of mutations. Although further investigations are needed to clarify the mechanisms downstream the activation of M_2_ receptors, the reported data point to M2 receptors as promising new therapeutic tools in glioma therapy as well as for other pathologies where dysfunction of M2 activity are involved (*e.g*. Chagas' disease) 45. Currently, drugs aimed to increase the levels of ACh are already in use or under clinical trials in pathologies characterized by ACh low levels; this is the case for acetylcholinesterase inhibitors in AD, selective muscarinic agonists such as alvameline and cevimeline (M1 receptor) for the treatment of AD or Sjogren's disesase, nicotinic and muscarinic receptors selective antagonists, such as darifenacin (M3 antagonist) or iprotropium (M1/M3 antagonist) have also been proposed for the treatment of overactive blabber, and chronic obstructive pulmonary disease (COPD) 12, 46. In conclusion, our results offer an important contribution to the discussion on modulation of cholinergic system activity in different contexts (cancer, inflammation, neurological disease….) and encourage searching for novel drugs with high selectivity and reduced side effects that may be resolved working on the chemical nature of the compounds.

## Supporting information

**Figure S1** Immunohistochemistry of M2 receptors in fresh glioma biopsy. The arrows indicated the positive cells (1000X).

**Figure S2** Immunocytochemistry analysis of M2 receptor in U251 cells after siRNA transfection. (A) Control (in absence of siRNA). The red arrows indicate the positive cells (150x). (B) Hoechst staining of U251 siRNA transfected cells; the blue arrows indicate the nuclei of cells that are completely negative for M2 staining in Fig. C (150x). (C) M2 staining in U251 siRNA transfected cells (150x). The immunostaining appears decreased respect to the control (A); some spots of fluorescence appear on the plasma membrane of the cells, as evident in the frame showing higher magnification of one cell (300x).
