# Supplementary figures and images for: M2 receptor activation inhibits cell cycle progression and survival in human glioblastoma cells
Source: J Cell Mol Med. 2013 Mar 14;17(4):552–66. doi: 10.1111/jcmm.12038 (PMC3822656; doi:10.1111/jcmm.12038)

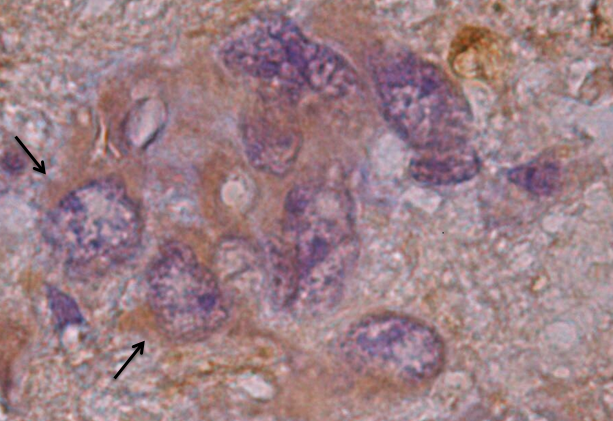

Supplement: Supplementary file 1 [file jcmm0017-0552-SD1.tif]

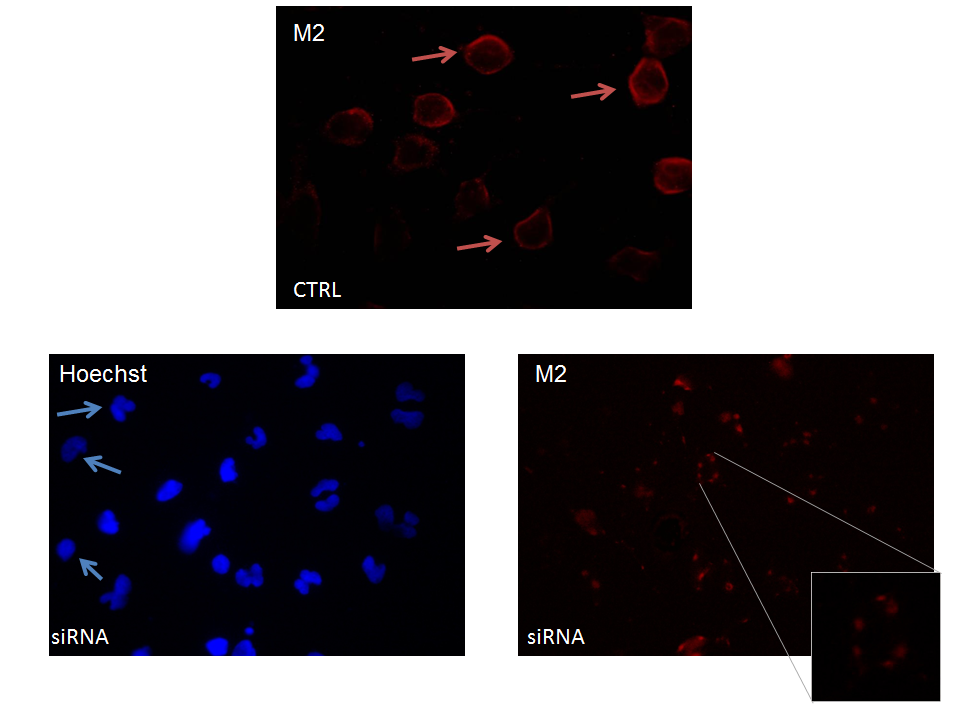

Supplement: Supplementary file 2 [file jcmm0017-0552-SD2.tif]
